# The Edinburgh Postpartum Depression Scale: Stable structure but subscale of limited value to detect anxiety

**DOI:** 10.1371/journal.pone.0221894

**Published:** 2019-09-09

**Authors:** Angarath I. van der Zee-van den Berg, Magda M. Boere-Boonekamp, Catharina G. M. Groothuis-Oudshoorn, Sijmen A. Reijneveld

**Affiliations:** 1 Department of Health Technology and Services Research, Technical Medical Centre, University of Twente, Enschede, the Netherlands; 2 Department of Health Sciences, University Medical Center Groningen, University of Groningen, Groningen, the Netherlands; University of Oslo, NORWAY

## Abstract

**Purpose:**

The Edinburgh Postnatal Depression Scale (EPDS) aims at detecting postpartum depression. It has been hypothesized that a subscale (items 3, 4, 5) may detect anxiety. The aim of this study is to assess whether this EPDS anxiety subscale is present in a community-based dataset, and if so, to assess its validity and stability during the first six months postpartum.

**Methods:**

We obtained EPDS data of a community sample of 1612 women at 1 month, with follow-up at 3 and 6 months, postpartum (Post-Up study). We performed an exploratory factor analysis on the EPDS forcing two- and three-factor solutions. We assessed the correlations of the extracted factor subscales and the total EPDS with the short-form of the STAI (STAI-6). We examined the stability of the identified factors by means of a confirmatory factor analysis (CFA), using the EPDS data collected at 3 and 6 months postpartum.

**Results:**

Both the two- and three-factor solutions contained a hypothesized anxiety subscale of items 3,4,5,10, and fitted well with the 3- and 6-months EPDS data, with CFI and TLI values >.99 and RMSEA and SRMR values < .035 and < .45. The subscale’s Pearson correlations with the STAI-6 were moderate: .516, compared to .643 for the total EPDS.

**Conclusions:**

The factor structure of the EPDS is stable across the first six months postpartum, and includes the subscale assumed to represent anxiety. However, this subscale as well as the total EPDS correlate only moderately with anxiety criteria. Using the EPDS thus does not imply adequate screening for anxiety.

## Introduction

In the postpartum period, both depression and anxiety frequently occur, with reported meta-analysis period prevalence rates of 19.2% for major and minor depression (0–3 months postpartum) [1], and 13.2% for anxiety (0–24 weeks postpartum) [2]. The co-occurrence of depression and anxiety seems to be high; Fallah-Hassani et al. reported meta-analysis prevalence rates of 3.5 to 9.2% in the first 24 weeks postpartum [3]. Comorbidity of depression and anxiety is associated with more persistent depression [4, 5], which increases the risk of negative consequences for the offspring [6, 7]. Therefore, adequate recognition and treatment of both depression and anxiety are essential. However, until now interventions focusing on postpartum maternal mental wellbeing have mainly addressed postpartum depression (PPD) [8].

A key step in addressing maternal mental disorders in the postpartum period is early detection. Primary care settings usually make use of the Edinburg Postnatal Depression Scale (EPDS) [9] to screen for PPD [10]. Though the EPDS was developed to detect PPD, many studies of its structure detect two or three factors, recently summarized in an overview by Coates et al. and Kozinsky et al. [11, 12]. Interestingly, the majority of the factor solutions found contained a subscale formed by three items (3, 4 and 5), interpreted as being an anxiety subscale, even though evidence on the total number of factors and item allocation is inconclusive. This hypothesized anxiety subscale, named the EPDS-3A by Matthey [13], might be of clinical interest when considering screening for anxiety along with PPD. However, evidence for the validity of the EPDS-3A to detect anxiety is limited, provided by studies with small or selected populations [13–15]. The same limited evidence applies to the postpartum stability of the subscale, with only one study in a community based sample [12] finding a stable structure at two postpartum intervals, thereby making conclusions on clinical use rather premature. Therefore, the aim of this study is to assess whether the hypothesized EPDS anxiety subscale is present in EPDS data of a large community based sample, and if so, to assess whether this subscale enables measurement of anxiety in addition to depression, and is stable across the first six months postpartum.

## Materials and methods

### Procedures and sample

We used data of the Post-up study, a study on the effectiveness of repeated screening for PPD with the EPDS, compared to care-as-usual in well-child care. The current study was limited to data on the intervention region. Procedures, including details on enrollment and exclusion criteria and on data collection, are fully described elsewhere [16]. In the intervention region, 4275 women with a newborn child visiting the participating well-child care centers in the inclusion period were eligible for enrollment. Informed consent was obtained from 2265 mothers, of whom 1843 completed the baseline assessment (3 weeks postpartum). Prior to their visit to the well-child care center at 1, 3 and 6 months, intervention mothers were asked to fill in a hardcopy version of the EPDS. During their consultations, well-child care professionals used the EPDS results, and afterwards returned the anonymized EPDS forms to the research team for further analysis. Data of mothers with a completed baseline assessment and at least one EPDS returned were used in this study, resulting in a sample of 1612 women, i.e. a retention of 71.1%.

### Measures

The Edinburg Postnatal Depression Scale is a 10-item self-report measure, developed specifically for use in community samples of postpartum mothers [9]. By choosing one of four responses (scored 0 to 3), women can indicate the extent to which each statement corresponds to their mood over the past 7 days. The sum of item scores forms the total score, with higher scores implying more depressive symptoms. The Dutch version was validated in 1992 [17], showing adequate concurrent validity, and a standardized Cronbach’s alpha of .82.

Anxiety level was measured at baseline assessment at 3 weeks postpartum with the 6-item short form of the state scale of the Spielberger State-Trait Anxiety Inventory (STAI-6) [18]. For each item (calm, tense, upset, relaxed, content and worried) the experienced current status is indicated on a 4-point scale. The Dutch version has been shown to have good reliability (Cronbach’s alpha .83) and validity (correlation with the STAI full version: .95) [19].

Background characteristics, measured at 3 weeks post-partum, concerned demographic characteristics of the mother (age, native country, living in an urban area, educational level, employment, single mother); pregnancy characteristics (complications, preterm birth, firstborn); history of depression; and breastfeeding of the child.

### Statistical analysis

First, we described the sample. Second, we examined the suitability of our data for structure detection by performing the Kaiser-Meyer-Olkin Measure of Sampling Adequacy test (KMO) and Bartlett’s test of sphericity. Third, we assessed the factor structure of the EPDS and whether in mothers one month post-partum we could indeed identify an anxiety subscale, in addition to a depression subscale. We did so by assessing the factor structure of the EPDS, using an Exploratory Factor Analysis (EFA) with maximum likelihood extraction and oblique rotation (direct oblimin) [20, 21], based on a polychoric covariance matrix. In this EFA we forced two- and three-factor solutions as parallel analyses. We used a polychoric correlation matrix because of the skewness of distribution of answer categories of the EPDS items. We evaluated the EFAs based on eigenvalues, total amount of variance explained, factor loading and Cronbach’s alpha.

Fourth, we assessed whether one of the extracted factor subscales indeed measured anxiety, by calculating the Pearson correlations of the subscale scores and of the total EPDS score with the STAI-6. In addition we computed the area under the receiver operating characteristic (ROC) curve (AUC) for both the anxiety-subscale and the total EPDS scores, with the STAI-6 (cut-off ≥ 42, prorated score) [22, 23].

Finally, we assessed the stability of the EPDS structure, i.e. its measuring of both depression and anxiety, across the first six months postpartum. We did so by determining whether the structure of the EPDS at 3 and 6 months differed from that at 1 month, using CFA. Items were fixed on the factor with the highest loading. Fit indices reported are Chi-square (including (df) and *p*), the comparative fit index (CFI), the Tucker-Lewis fit index (TLI), the root mean square error of approximation (RMSEA) (including 90% confidence interval (CI) and *p*) and the Standardized Root Mean Square Residual (SRMR). CFI and TLI values greater than .95, RMSEA < .06 and SRMR < .08, were considered indicative of good fit, preferably in combination [24, 25]. We performed data analyses using SPSS 24 and R with the lavaan package [26].

## Results

### Background characteristics

Background characteristics of the sample are presented in Table 1. National demographic data of the Dutch population of 2013 show comparable characteristics for mean age at giving birth (31.0 years), first-born child (46%) and medium-high education (84.7% for all women aged 25 to 45 years)[27].

**Table 1:** Background characteristics (mean plus standard deviation (SD), or %) of participants .

Background characteristic	Participating Mothers (N = 1612)
Age mothers (mean)	30.6 (SD = 4.0)
Age partners (mean)	33.5 (SD = 4.8)
Mother Dutch born	95.8%
Partner Dutch born	96.0%
Single mother	1.0%
Living in urban area*	11.5%
Mother education** (medium-high)	88.8%
Partner education** (medium-high)	81.6%
Mother employed (>12hours/week)	83.0%
Partner employed (>12hours/week)	94.5%
Depression	
• Lifetime	17.7%
• During pregnancy	1.6%
• Previous postpartum ***	4.1%
First-born child	45.0%
Preterm birth****	3.6%
Complications during pregnancy	24.6%
Breastfeeding started after birth	74.4%

* ≥ 1000 addresses/km2;

** comparable with level 3 or higher of the ISCED classification (International Standard Classification of Education)[28]

*** percentage of whole sample;

**** birth before 37 weeks gestation

Of the total sample of 1612 mothers, of whom at least one EPDS had been returned to the research team, 1339 mothers filled in an EPDS at 1 month (SD 1.1 weeks), 1272 at 3 months (SD 1.7 weeks) and 1040 at 6 months (SD 1.5 weeks). Mean EPDS scores were 3.7 at 1 month, 2.8 at 3 months and 2.7 at 6 months.

### Factor structure of EPDS at one month post-partum

The EPDS data at one month postpartum were found suitable for factor analysis with a KMO statistic of .91 and a significant Bartlett’s test of sphericity (p< 0.001). Table 2 shows the outcomes of the EFA with forced two -and three-factor solutions. Both the two- and three-factor solutions resulted in a factor formed by items 3, 4, 5 and 10, labeled as ‘anxiety subscale’. In the two-factor solution the other factor was formed by the remaining items 1, 2, 6, 7, 8, 9, labeled ‘two-factor depression subscale’. In the three-factor solution these items were split up in a subscale formed by items 1 and 2, labeled the ‘three-factor anhedonia subscale’, and a subscale formed by items 6,7, 8, 9, labeled the ‘three-factor depression subscale’. In both solutions item 10 presented with low loadings and minimal cross loadings. This was also the case for item 6 in the three-factor solution. Eigenvalues ranged from 1.85 to 3.90, and resulted in a total variance explained of 60.7% for the two-factor solution and 64.4% for the three-factor solution. Cronbach’s alphas for the two- and three-factor solutions varied from .61 to .79, implying acceptable reliability. Correlations between the factors in the factor models can be found in S1 and S2 Figs.

**Table 2:** Factor solutions of items of the Edinburgh Postpartum Depression Scale for the forced two- and three-factor solutions at 1 month: Factor loading, Eigenvalues, variances explained and Cronbach’s alphas.

	Two-factor			Three-factor			
EPDS items	1	2		1	2	3	
1. I have been able to laugh and see the funny side of things	**.822**	.004		.032	.094	**.821**	
2. I have looked forward with enjoyment to things	**.873**	.168		.096	.065	**.786**	
3. I have blamed myself unnecessarily when things went wrong	-.032	**.766**		.244	**.647**	-.168	
4. I have been anxious or worried for no very good reason	.060	**.623**		.092	**.587**	.030	
5. I have felt scared or panicky for no good reason	.023	**.760**		.113	**.826**	.142	
6. Things have been getting on top of me	**.611**	.213		**.330**	.221	.325	
7. I have been so unhappy that I have had difficulty sleeping	**.622**	.245		**.510**	.245	.152	
8. I have felt sad or miserable	**.859**	.003		**.771**	-.036	.155	
9. I have been so unhappy that I have been crying	**.795**	.093		**.888**	.007	.015	
10. The thought of harming myself has occurred to me	.303	**.482**		.375	**.421**	.003	
Eigenvalues	3.90	2.17		2.52	2.07	1.85	
Variance explained %	39.04%	1.67%	60.71%*	25.21%	20.71%	18.46%	64.39%*
Cronbach’s alpha	0.79	0.61		0.73	0.61	0.67	

Extraction Method: Maximum Likelihood. Rotation Method: Oblimin with Kaiser Normalization.

Rotation converged in 20 iterations.

* Total variance explained

### Correlations of total EPDS and subscales with STAI-6

The correlation with the STAI-6 (maximum administration interval of 7 days (N = 550)) was strongest for the total EPDS (Pearson correlation .643). Moreover, the correlation of the STAI-6 with the two-factor depression subscale was stronger (.605) than the correlation with the anxiety subscale (.516). The three-factor subscales resulted in correlations with the STAI-6 of .520 (anhedonia subscale) and .565 (depression subscale). Similar correlations resulted from including more mothers by enlarging the maximum administration interval between EPDS and STAI-6 to 7 weeks (N = 1256), and from leaving item 10 out of the anxiety subscale. AUC for the anxiety-subscale was .729 versus .811 for the total EPDS.

### Stability of EPDS structure across the first six months postpartum

Table 3 shows the extent to which the two- and three-factor models fit the EPDS data collected at three and six months postpartum. CFI and TLI values > .99 and RMSEA and SRMR values < .035 and < .45 respectively, indicate good fit for both models. The three-factor model found in the EFA performed the best. Omitting item 10 out from the CFA resulted in comparable outcomes (S1 Table).

**Table 3:** Fit indices corresponding with the confirmatory factor analysis of the two- and three- factor model for 3 and 6 months.

Fit indices	3 months	3 months	6 months	6 months
	two-factor model	three-factor model	two-factor model	three-factor model
Chi square	73.8	29.6	71.8	47.1
(df)	34	32	34	32
*p* (Chi square)	< .001	0.587	< .001	< .001
RMSEA (90%)	.030 (.021-.040)	.000 (.000-.019)	.033 (.022-.043)	.021 (.004-.034)
p-value	100%	100%	99.7%	100%
RMSEA < = .05				
CFI	.997	1.0	.997	.999
TLI	.996	1.0	.996	.998
SRMR	.039	.023	.044	.034

## Discussion

During our factor structure analysis of the EPDS data, collected in a large community sample of postpartum women, we found the EPDS to have a subscale formed by items 3, 4, 5 and 10, in both the two- and three-factor solutions. This hypothesized anxiety subscale was stable across the first six months postpartum. We further found only a moderate correlation of this subscale with the STAI-6 as criterion for anxiety, at one month postpartum. Correlations with the STAI-6 were stronger, though still moderate, for the total EPDS, and also for the depression subscale from both the two- and three-factor solutions.

### Findings compared to current evidence

The presence of a subscale containing EPDS items 3, 4 and 5 in our EPDS factor structure analysis confirms previous findings from comparable studies with a large community sample and timing of the EPDS within 4–6 weeks postpartum [12, 29–32]. Our finding also confirms findings from studies with broader or different postpartum timeframes or more specific populations [13, 14, 33, 34]. Our study results differ from these studies regarding the position of item 10 (the item asking for suicidal ideation), as in most studies item 10 is loading more on the depression factor. Our inclusion of item 10 in the anxiety subscale may have been caused by our use of a polychoric matrix, which may better suit the data concerned. However, as in previous studies, loadings of item 10 were low, i.e. the item was rather undetermined. This may align with the vision to consider item 10 as an item with the specific function to detect potential suicidal risk. Regarding the stability of the EPDS in the postpartum period, our findings correspond to the outcomes of Coates et al. [12], who found a stable structure with the hypothesized anxiety subscale, from 8 weeks to 8 months postpartum. In sum, the hypothesized anxiety subscale appears to be present and stable in large community samples.

Our findings on the correlation of the hypothesized anxiety subscale are in line with the study of Brouwers et al. [35], who also found moderate correlations during pregnancy for the anxiety subscale and the STAI, and somewhat stronger correlations for the total EPDS as well as the depression subscale. Other studies assessing only correlations between the STAI (full-form) and the total EPDS, reported substantially stronger correlations [36–38]. Two studies with positive conclusions on the value of using the anxiety subscale to detect anxiety did not validate the subscale [39, 40]. The only study providing evidence in favor of the validity of a 3, 4, 5 item anxiety subscale was that of Matthey [13] (N = 238, 7.6%, met the anxiety disorder criteria), with a subscale sensitivity of 67% and a specificity of 82% at 6 weeks postpartum (criterion Diagnostic Interview Schedule).

The limited evidence for the hypothesized subscale’s representation of anxiety might imply that this subscale actually does not represent anxiety. Brouwers et al. [35] noted the subjective, negative judgement, incorporated in items 3, 4 and 5 (e.g. “for no good reason”), which may relate to another construct like low self-esteem. The correlations of the total EPDS and other subscales with anxiety, indicate that anxiety is measured at least as much by the other EPDS-items. This implies that the total EPDS does to some extent detect anxiety symptoms in addition to depression symptoms, but that its subscales do not have added value for this.

### Strengths and limitations

Strengths of our study are its community based sample and its large sample size. Another strength is our use in the analyses of a polychoric matrix, which is a more adequate statistical method when performing a factor analysis with ordinal data [41], but as yet rarely used in factor analyses of the EPDS.

A limitation of our study might be the use of the STAI-6 as anxiety criterion, as it probably measures depression in addition to anxiety, as is similar to the STAI full form [42, 43]. Further, the non-simultaneous administration of the EPDS and STAI-6 may have deflated the correlations, though in our analyses we minimized this effect by limiting the maximum interval to 7 days.

### Implications

Our study provides clear evidence for an EPDS subscale of items 3, 4, 5 and 10 which is stable across the first six months postpartum, but could not ascertain this subscale to adequately detect anxiety symptoms. The total EPDS performed better than our hypothesized anxiety subscale, but still correlates only moderately with our anxiety measure. This implies that using the EPDS in routine care, does not enable the professional to detect most cases of both depression and anxiety, nor enables to discriminate between the two. Research findings based on the EPDS subscales should be interpreted with caution.

Further research is needed to assess the maximum potential of the EPDS in the detection of anxiety, and whether additional efforts should be made to detect both depression and anxiety reliably and efficiently in an early stage. This may add to screening policies for both depression and anxiety regarding women during pregnancy and the postpartum period [44, 45], and thus promote maternal mental health.

## Conclusion

Our large community based study shows that the factor structure of the EPDS is stable across the first six months postpartum and includes a subscale generally assumed to represent anxiety. This subscale correlates only moderately with our anxiety criterion though, with the total EPDS performing slightly better. Adequate screening for anxiety may require an additional effort on top of the current EPDS.

### Ethical approval

All procedures performed in studies involving human participants were in accordance with the ethical standards of the institutional and/or national research committee and with the 1964 Helsinki declaration and its later amendments or comparable ethical standards.

## Supporting information

S1 Fig Correlations two-factor model. (DOCX)

S2 Fig Correlations three-factor model. (DOCX)

S1 Table Fit indices corresponding with the Confirmatory Factor Analysis leaving item 10 out of the two- and three- factor model for 3 and 6. (DOCX)
