# Supplementary figures and images for: The Edinburgh Postpartum Depression Scale: Stable structure but subscale of limited value to detect anxiety
Source: PLoS One. 2019 Sep 9;14(9):e0221894. doi: 10.1371/journal.pone.0221894 (PMC6733480; doi:10.1371/journal.pone.0221894)

**S1 Fig Correlations two-factor model**


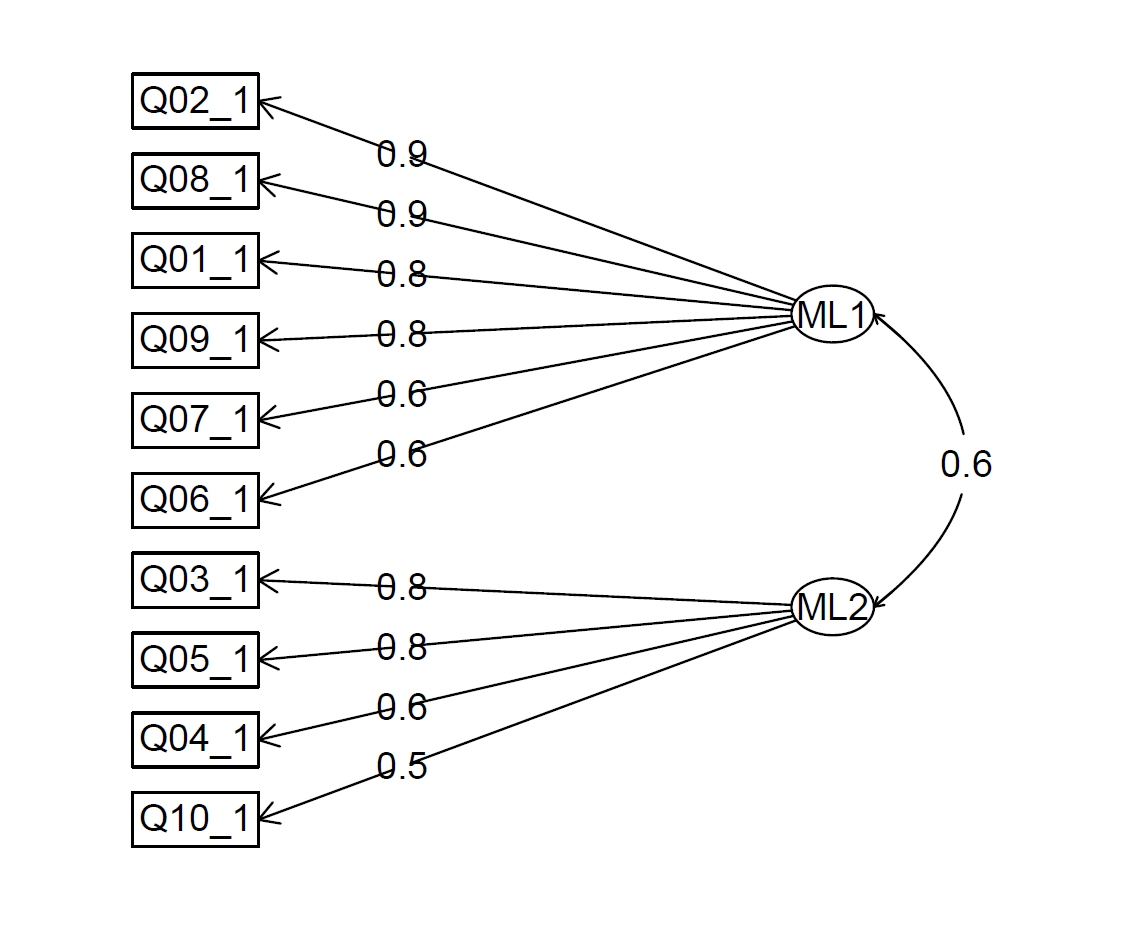

Supplement: S1 Fig — (DOCX) [file pone.0221894.s001.docx]

**S2 Fig Correlations three-factor model**


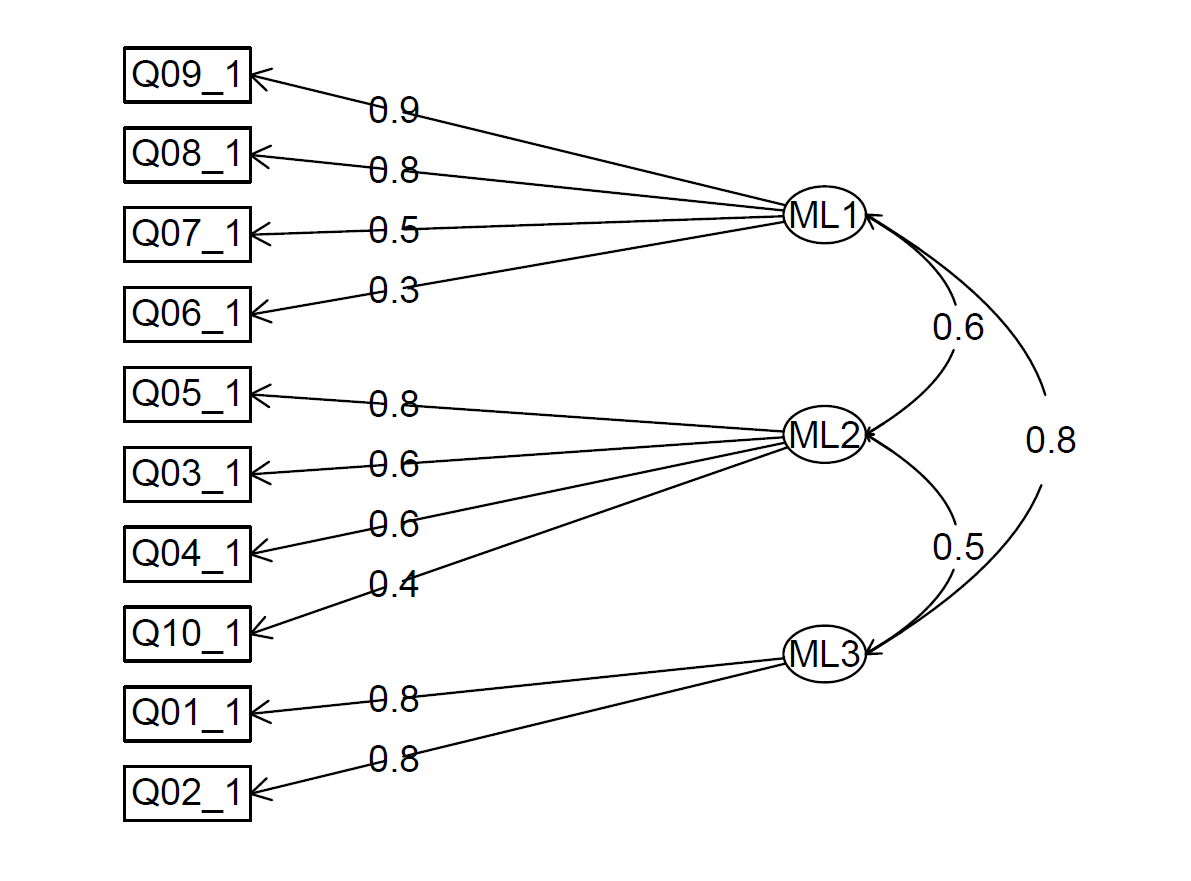

Supplement: S2 Fig — (DOCX) [file pone.0221894.s002.docx]
