# Supplementary material for: The Edinburgh Postpartum Depression Scale: Stable structure but subscale of limited value to detect anxiety
Source: PLoS One. 2019 Sep 9;14(9):e0221894. doi: 10.1371/journal.pone.0221894 (PMC6733480; doi:10.1371/journal.pone.0221894)
Supplement: S1 Table — (DOCX) [file pone.0221894.s003.docx]

**S1 Table Fit indices corresponding with the Confirmatory Factor Analysis leaving item 10 out of the two- and three- factor model for 3 and 6 months**

| Fit indices | 3 months | 3 months | 6 months | 6 months |
| --- | --- | --- | --- | --- |
|  | two-factor model | three-factor model | two-factor model | three-factor model |
| Chi square | 65.1 | 20.1 | 71.8 | 47.1 |
| (df) | 26 | 24 | 34 | 45 |
| *p* (Chi square) | <.001 | .691 | <.001 | <.001 |
| RMSEA (90%) | .035 (.024-.045) | 0.000 (0.000-0.018) | .033 (.022-.043) | .021 (.004-.034) |
| p-value | 99.3% | 100% | 99.7% | 100% |
| RMSEA <= .05 |  |  |  |  |
| CFI | .997 | 1.000 | .997 | .999 |
| TLI | .996 | 1.000 | .996 | .998 |
| SRMR | .042 | .023 | .044 | .034 |
